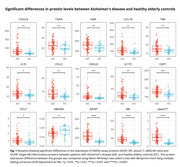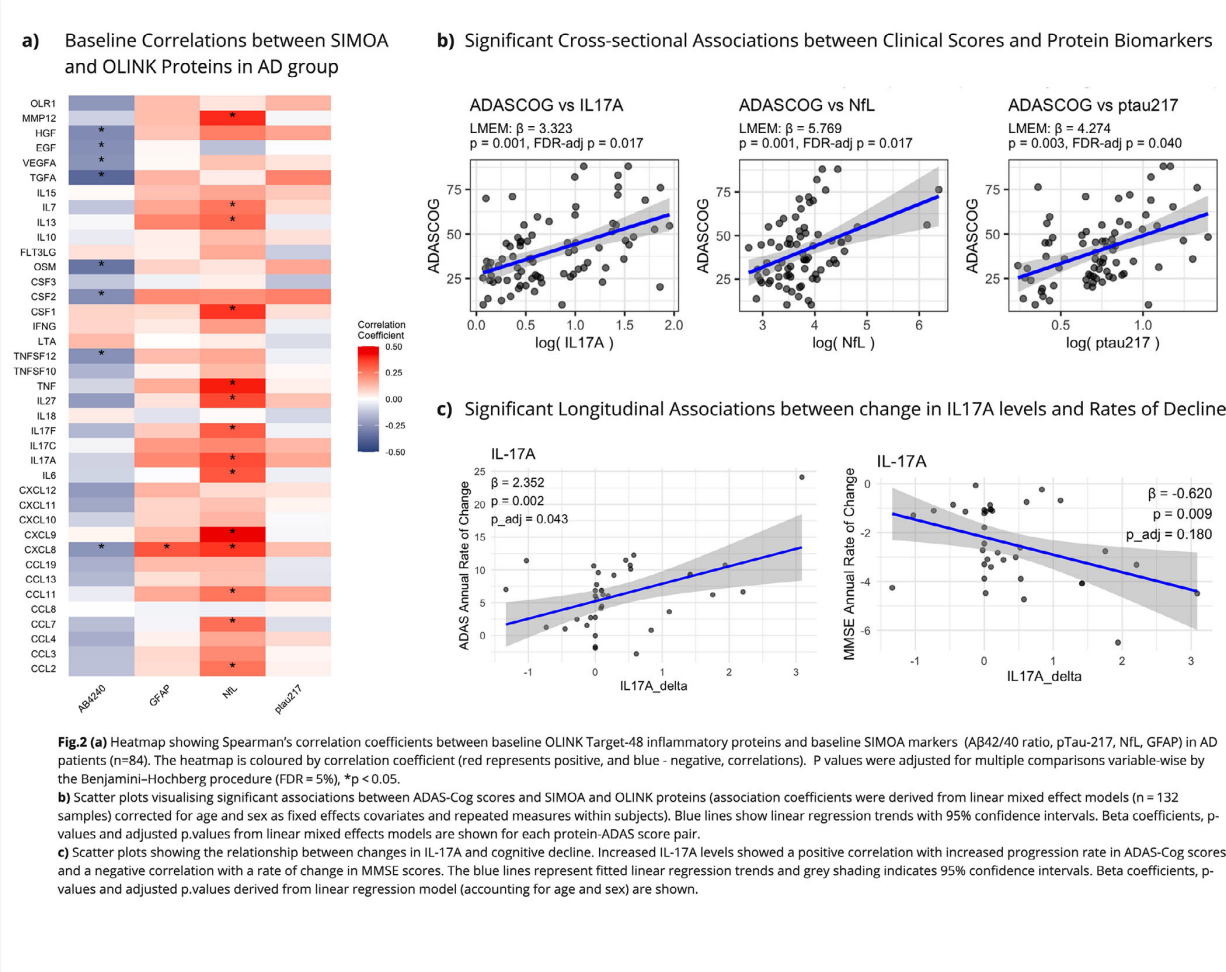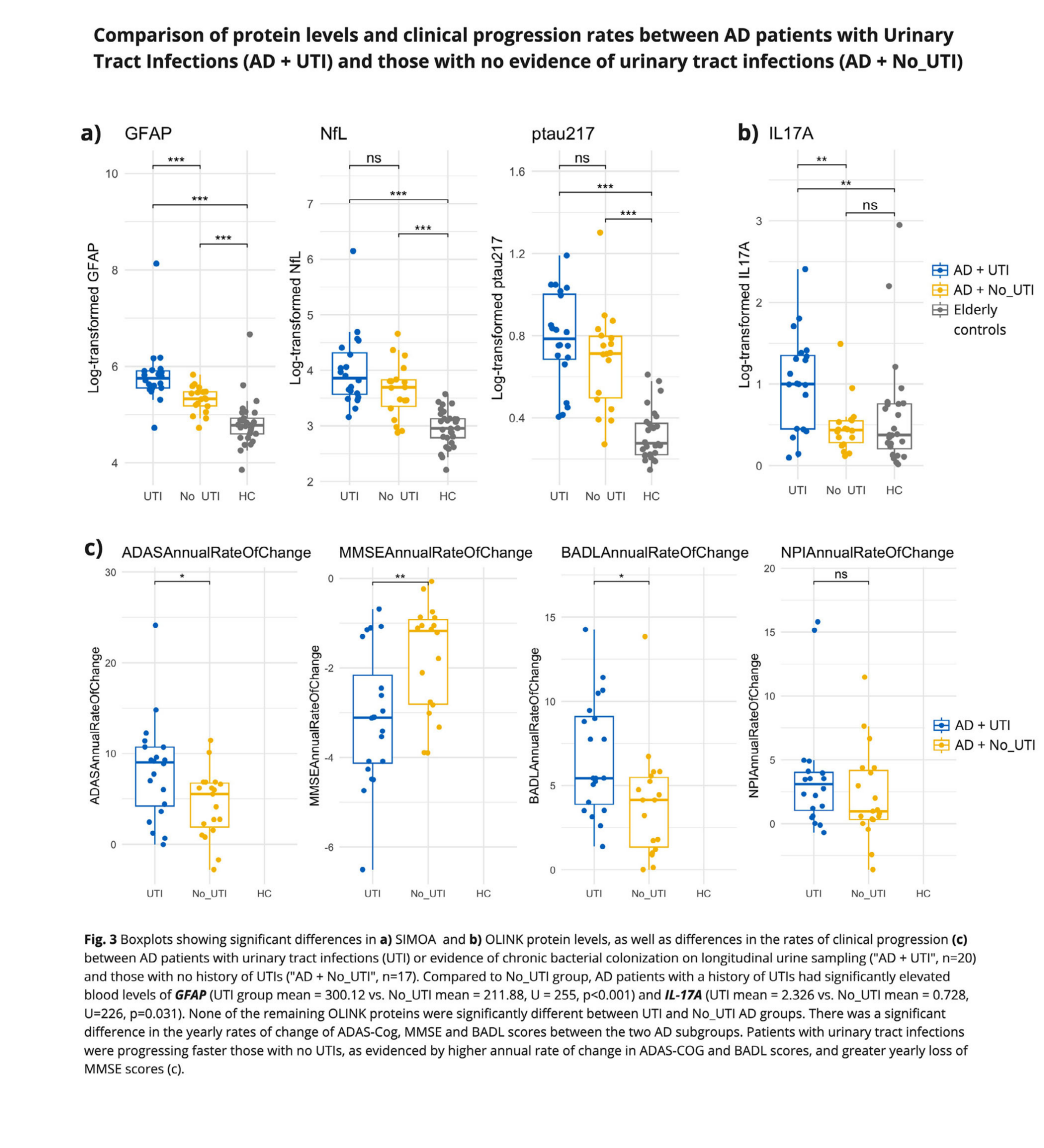# Relationships between inflammatory cytokines, neurodegenerative biomarkers, clinical progression and systemic infection in patients with Alzheimer's disease

**DOI:** 10.1002/alz70856_106435

**Published:** 2026-01-10

**Authors:** Magdalena A. Kolanko, Raphaella Jackson, Eyal Soreq, Michael CB David, Kirsten Jensen, Amanda J Heslegrave, Martin Tran, Michael Crone, Sarah Daniels, David Wingfield, Henrik Zetterberg, Paul Freemont, David J Sharp

**Affiliations:** ^1^ UK Dementia Research Institute Centre for Care Research and Technology, London, United Kingdom; ^2^ Imperial College London, Department of Brain Sciences, London, United Kingdom; ^3^ UK Dementia Research Institute Centre for Care Research and Technology, London, England, United Kingdom; ^4^ UK Dementia Research Institute, Care Research and Technology Centre, Imperial College London, London, United Kingdom; ^5^ Department of Brain Sciences, Imperial College London, London, United Kingdom; ^6^ Imperial College London, London, United Kingdom; ^7^ UK Dementia Research Institute Centre for Care Research and TechnologyUK Dementia Research Institute Centre for Care Research and Technology, London, England, United Kingdom; ^8^ Imperial College London, London, England, United Kingdom; ^9^ Department of Neurodegenerative Disease, UCL Queen Square Institute of Neurology, London, United Kingdom; ^10^ UK Dementia Research Institute at UCL, London, United Kingdom; ^11^ UK Dementia Research Institute, Care Research and Technology Centre, London, United Kingdom; ^12^ Department of Neurodegenerative Disease and UK Dementia Research Institute, UCL Institute of Neurology, Queen Square, London, United Kingdom; ^13^ Institute of Neuroscience and Physiology, The Sahlgrenska Academy at University of Gothenburg, Mölndal, Sweden

## Abstract

**Background:**

Systemic infection such as urinary tract infection (UTI) causes delirium and faster cognitive decline in patients with Alzheimer's disease (AD). Changes in brain cytokine levels in response to systemic infection influence amyloid‐β, tau and glial pathology and exacerbate cerebrovascular dysfunction in animal models. Here we investigate the relationships between blood inflammatory and neurodegenerative markers, urinary tract infections and disease progression in people living with AD.

**Method:**

We analysed longitudinal blood samples from 84 AD patients and 29 elderly controls using two platforms: OLINK®Target‐48 Inflammation and ultrasensitive single‐molecule array (Simoa®) assay. We stratified AD patients according to the presence/absence of UTIs confirmed on longitudinal urine sampling for urine microscopy and culture. Repeated ADAS‐COG, NPI and BADL were used to assess disease progression.

**Result:**

AD patients had elevated NfL, GFAP, *p*‐tau217, as well as a range of inflammatory proteins (Figure 1). NfL, a marker of axonal injury, correlated with multiple cytokines in AD group (Figure 2a), including IL‐17A, which was also associated with cognitive performance measured by ADAS‐COG in cross‐sectional and longitudinal analyses (Figure 2b,c). The presence of urinary tract infections was associated with higher GFAP (Figure 3a), elevated IL‐17A (Figure 3b) and faster rates of cognitive decline on MMSE, ADAS‐COG, and BADL scales (mean MMSE score change = ‐3.11points/year (SD=1.5) in UTI group versus ‐1.77 points/year (SD=1.22) in No_UTI group, U=84, *p* = 0.005).

**Conclusion:**

We show that blood inflammatory cytokines are elevated in AD, correlate with NfL plasma levels and cognitive performance, and are influenced by urinary tract infections. AD patients with chronic UTIs have elevated levels of GFAP and IL‐17A and progress faster than those with no history of UTIs. Recent AD mouse models support the mechanistic link between IL‐17A accumulation, cognitive deficits and neurodegeneration. These findings highlight systemic infection as an important contributor to dementia, requiring early identification and treatment in this vulnerable population.